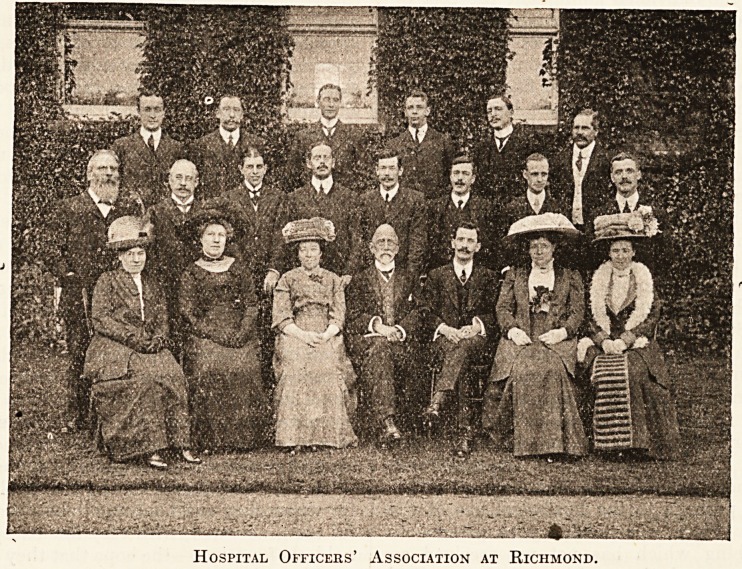# Hospital Officers' Association Visit to Richmond

**Published:** 1911-07-15

**Authors:** 


### HOSPITAL OFFICERS' ASSOCIATION VISIT TO RICHMOND.

Mr. Richard Allen, the secretary of the Royal Hospital, Richmond, has had the pleasure of welcoming thirty members of the above association, who, with their friends, visited the hospital and explored the borough. Mr. Allen, who, we believe, has not yet published his history of the Royal Hospital, delivered an historical address on the institution from its early days as a cottage hospital to its present position with some seventy beds. The cottage was the home of Thomson, whose "Seasons" and "Castle of Indolence" are read still by the few people who read poetry, and what is now the entrance hall formed the two ground-floor rooms, in one of which Thomson died in 1748. Mr. George Ross, Thomson's friend, then bought and "improved" the cottage, which became Rosedale House as the property of Mrs. Boscowen. In 1805 Lord Shaftesbury bought it, and his Countess lived there for nearly half a century. Finally, in 1863 £40 over from the children's feast held in celebration of the marriage of King Edward and Queen Alexandra was made the beginning of the fund out of which the present hospital has sprung. The actual institution was provided in 1867. In 1882 two wards were added, and in 1894 a new ward-room, a ward for children, new rooms for the nurses and servants were built. Ten years later an out-patients' department was added, and in 1908 two small wards of six beds each for men and women were given by Mrs. Swan, of Teddington. Mr. Andree, the assistant secretary, then helped to show the visitors round the hospital, whose only regret was the absence of Mr. W. Sandover, the chairman of the ward. Mr. A. Watkins, in the name of the association, thanked Mr. Allen and Mr. Andree for their kindness, and the day concluded with a visit to the places of interest in the town. If this afternoon merely produces in the secretaries present a determination to work out the histories of their own hospitals it will have done an exceedingly useful work. A little trouble, as in the case of the Royal Hospital, Richmond, will produce a record that is full of interest, and Mr. Allen has set a capital example to all who had the pleasure of listening to him.

Hospital Officers' Association at Richmond.

Standing (left to right) top row: Mr. W. Pearson, Gravesend Hospital; Mr. W. Watkins, St. Bartholomew's; Mr. Bishop *; Mr. Dickinson *; Mr. A. J. Borer, Central London Ophthalmic Hospital; Mr. A. F. Andree, Royal Hospital, Richmond. Second row: Mr. J. Watts, Royal Hospital for Incurables; Mr. A. Watkins, St. Bartholomew's; Mr. J. H. Johnson, Royal Westminster Ophthalmic; Mr. A. E. Thomas, Hampstead General; Mr. Yates *; Mr. T. Smith, Royal Hospital, Richmond; Mr. F. P. Carroll, Charing Cross Hospital; Mr. Ian MacKay, Chelsea Hospital for Women.

Sitting: Mrs. Brown; Miss R. Watkins; Miss Watkins; Mr. T. F. Myers, late of Yarrow Convalescent Home, now retired; Mr. R. Allen, Royal Hospital, Richmond; Mrs. T. Smith; Mrs. R. Allen.

* These gentlemen were friends of the members.

## Figures and Tables

**Figure f1:**